# An integrated digital/clinical approach to smoking cessation in lung cancer screening: study protocol for a randomized controlled trial

**DOI:** 10.1186/s13063-017-2312-x

**Published:** 2017-11-28

**Authors:** Amanda L. Graham, Michael V. Burke, Megan A. Jacobs, Sarah Cha, Ivana T. Croghan, Darrell R. Schroeder, James P. Moriarty, Bijan J. Borah, Donna F. Rasmussen, M. Jody Brookover, Dale B. Suesse, David E. Midthun, J. Taylor Hays

**Affiliations:** 1 Schroeder Institute for Tobacco Research and Policy Studies at Truth Initiative, 900 G Street NW, 4th Floor, Washington, DC 20001 USA; 2 Department of Oncology, Georgetown University Medical Center/Cancer Prevention and Control Program, Lombardi Comprehensive Cancer Center, Washington, DC USA; 3 Mayo Clinic Nicotine Dependence Center, Mayo Clinic, Rochester, MN USA; 4 Division of Primary Care Internal Medicine, Mayo Clinic, Rochester, MN USA; 5 Division of Biomedical Statistics and Informatics, Department of Health Sciences Research, Mayo Clinic, Rochester, MN USA; 6 Kern Center for the Science of Health Care Delivery, Mayo Clinic, Rochester, MN USA; 7 Division of Health Care Policy and Research, Mayo Clinic, Rochester, MN USA; 8 Division of Research and Education Systems Support, Mayo Clinic, Rochester, MN USA; 9 Division of Pulmonary and Critical Care Medicine, Mayo Clinic, Rochester, MN USA; 10 Division of General Internal Medicine, Department of Medicine, Mayo Clinic, Rochester, MN USA

**Keywords:** Cancer screening, Lung cancer, Smoking cessation, Internet, Text messages, Tobacco use cessation, Counseling

## Abstract

**Background:**

Delivering effective tobacco dependence treatment that is feasible within lung cancer screening (LCS) programs is crucial for realizing the health benefits and cost savings of screening. Large-scale trials and systematic reviews have demonstrated that digital cessation interventions (i.e. web-based and text message) are effective, sustainable over the long-term, scalable, and cost-efficient. Use of digital technologies is commonplace among older adults, making this a feasible approach within LCS programs. Use of cessation treatment has been improved with models that proactively connect smokers to treatment rather than passive referrals. Proactive referral to cessation treatment has been advanced through healthcare systems changes such as modifying the electronic health record to automatically link smokers to treatment.

**Methods:**

This study evaluates the impact of a proactive enrollment strategy that links LCS-eligible smokers with an evidence-based intervention comprised of a web-based (WEB) program and integrated text messaging (TXT) in a three-arm randomized trial with repeated measures at one, three, six, and 12 months post randomization. The primary outcome is biochemically confirmed abstinence at 12 months post randomization. We will randomize 1650 smokers who present for a clinical LCS to: (1) a usual care control condition (UC) which consists of Ask–Advise–Refer; (2) a digital (WEB + TXT) cessation intervention; or (3) a digital cessation intervention combined with tobacco treatment specialist (TTS) counseling (WEB + TXT + TTS).

**Discussion:**

The scalability and sustainability of a digital intervention may represent the most cost-effective and feasible approach for LCS programs to proactively engage large numbers of smokers in effective cessation treatment. We will also evaluate the impact and cost-effectiveness of adding proven clinical intervention provided by a TTS. We expect that a combined digital/clinical intervention will yield higher quit rates than digital alone, but that it may not be as cost-effective or feasible for LCS programs to implement. This study is innovative in its use of interoperable, digital technologies to deliver a sustainable, scalable, high-impact cessation intervention and to facilitate its integration within clinical practice. It will add to the growing knowledge base about the overall effectiveness of digital interventions and their role in the healthcare delivery system.

**Trial registration:**

ClinicalTrials.gov, NCT03084835. Registered on 9 March 2017.

**Electronic supplementary material:**

The online version of this article (doi:10.1186/s13063-017-2312-x) contains supplementary material, which is available to authorized users.

## Background

Lung cancer screening (LCS) provides a unique opportunity to deliver tobacco dependence treatment on a population-wide basis. More than 4 million current smokers age 55+ years are likely to be eligible for LCS [1, 2]. LCS saves lives through early identification of lung cancer. However, given that most lung screens are negative [2] and that smoking causes 11 other types of cancer as well as cardiovascular disease and other lung disease [3], the health benefits of providing effective tobacco dependence treatment for LCS patients are far-reaching. Smokers seeking LCS report interest in quitting and receiving cessation services [4–8] and simulation models predict that tobacco dependence treatment can improve the cost-effectiveness of LCS by 20–50% [9, 10]. Delivering tobacco dependence treatment that is feasible within a LCS program is crucial for maximizing the health benefits of screening [11, 12].

The Centers for Medicare and Medicaid Services (CMS) requires that LCS include a shared decision-making visit with smokers that includes “counseling on the importance of smoking cessation and furnishing of information about tobacco cessation interventions” [13]. This approach is analogous to the Ask–Advise–Refer (AAR) model of tobacco dependence treatment [6] in which healthcare providers ask about smoking status, advise a smoker to quit, and refer to readily available cessation treatment resources. A recent study by Ostroff et al. [14] reported that the majority of LCS programs “always” ask patients about smoking status (98.9%) and advise current smokers to quit (91.4%) but fewer reported delivery of cessation counseling (57%) or referral to a quit line (60.2%). Low rates of treatment referral in LCS programs are consistent with data from other healthcare settings [15–17]. Additionally, most smokers passively referred to treatment fail to follow through [15–18]. As noted by Fucito et al. [19], “patient motivation as a requirement for treatment (i.e. an opt-in model) means that most smokers will not receive smoking-cessation assistance” (page 9). Other treatment models are needed to address smoking cessation in the LCS setting.

Use of cessation treatment has been improved with models that proactively connect smokers to treatment resources (i.e. an opt-out model) rather than simply advising smokers to quit and providing referral information [20, 21]. Historically done through e-mail or fax, clinic staff sends a smoker’s contact information to a cessation treatment program – most often a quit line – followed by attempts by the cessation program to proactively reach the smoker. More recently, treatment uptake and engagement have been advanced through healthcare systems changes such as modifying the electronic health record (EHR) to automatically link smokers to tobacco dependence treatment [22–24]. Referred to as Ask–Advise–Connect (AAC), the healthcare provider asks about smoking status, advises a smoker to quit, and then immediately connects the smoker with cessation treatment by sending their contact information directly to a cessation program via an automated process through the EHR followed by proactive strategies by the program to engage the smoker in treatment. AAC is designed to address both clinic-level and patient-level barriers to treatment enrollment and has shown great promise. Compared to AAR, AAC yielded significant increases in quit line enrollment among smokers in a private healthcare system [25] and a safety net healthcare system [26].

The AAC approach combined with digital cessation interventions represents an exciting and novel approach to engaging smokers in treatment. The scalability and sustainability of a digital intervention may represent the most cost-effective and feasible approach for LCS programs to proactively engage large numbers of smokers in cessation treatment. Web-based (WEB) cessation programs are well-suited to deliver evidence-based cessation treatment. Problem-solving and skills-training information can be provided in interactive exercises; social support from peers and experts can be provided in an online community; and information and guidance about pharmacotherapy use can be provided. Unlike other treatment modalities, WEB interventions can be accessed 24/7 – particularly at relapse sensitive times – and used for as long as a smoker desires. The need for more extended and intensive cessation treatment strategies within the LCS setting was noted in a recent systematic review [27]. Large-scale trials of Internet interventions have reported quit rates of 18–20% at one year [28, 29] and several meta-analyses provide evidence of effectiveness, especially for programs that are interactive and tailored [30–33]. WEB cessation programs are cost-effective [34, 35] and scalable [35–38], able to accommodate thousands of additional users at little incremental cost. Evidence in support of AAC with digital interventions is beginning to emerge. Ray et al. [39] conducted a randomized trial in dental practices of electronic vs paper referral to an interactive, tailored cessation website. The proportion of smokers that registered on the website was fourfold higher among clinics using e-referral compared to clinics using paper referral. Abstinence rates were also significantly higher among e-referral clinics. The cost per registered smoker was in the range of $67–$87 [40], making this an extremely cost-efficient approach to connect smokers with evidence-based treatment. As a comparison, provider referrals using brochures yielded a cost of $597 per smoker recruited to a WEB intervention [41].

Text messaging (TXT) is also a proven cessation strategy [42]. Automated TXT programs can be designed to conform to established guidelines for smoking cessation [43] and can mirror elements of in-person counseling such as goal-setting and feedback, social support, and a personalized quit plan [44, 45]. TXT programs can be delivered long term [46, 47] and can increase the contact time and intensity of adjunct treatments [43]. The efficacy [48] and cost-effectiveness [49] of TXT programs for cessation are well-documented.

Current data and well-documented trends within healthcare substantiate the relevance of technology-based cessation interventions for older adults. Technology use is high and growing among older adults who are candidates for LCS (i.e. those aged 55 years and older [13]). Internet use and cell phone adoption among older adults continue to increase steadily: as of November 2016, 87% of those aged 50–64 years were Internet users, and 6/10 seniors (aged 65+ years) reported they go online [50]. The Internet is a trusted source of health information among older adults [51]. Approximately 74% of older adults (aged 60+ years) and 88% of Baby Boomers (aged 51–59 years) use a cellular device and an increasing number now have mobile Internet access [52]. Technology use among older adults is expected to become even more ubiquitous as the 78 million tech-savvy Baby Boomers grow older [53] and as national trends in healthcare and advances in health information technology continue to drive technology use among older adults [54–56].

Given that many patients seen in LCS programs will be older, long-term, heavy smokers with varying levels of interest in quitting [57], more intensive clinical intervention provided by a tobacco treatment specialist (TTS) may be necessary. TTS counseling has been integrated into a range of healthcare settings [58–60] and yielded quit rates in the range of 26.5–34% [43, 59–64]. A TTS working within a LCS program can provide more efficacious treatment than non-specialists or brief physician counseling [62, 65, 66]. Preliminary evidence suggests that implementing a TTS intervention before LCS may be more effective in promoting abstinence than interventions delivered after LCS. A pilot study by Ferketich et al. [67] reported six-month carbon monoxide (CO)-confirmed abstinence rates of 22% among smokers that received TTS counseling and pharmacotherapy before LCS compared with 11.1% among smokers that received the intervention after LCS. Treatment approaches that combine TTS counseling and digital interventions improve cessation outcomes and can be adopted in different types of healthcare systems [68]. Several large-scale studies have found that TTS counseling combined with a WEB cessation intervention yields significantly higher quit rates than a WEB intervention alone [29, 69]. To date, we are not aware of any studies that have evaluated proactive enrollment into a combined digital and clinical cessation intervention in LCS programs.

Building on the evidence in support of AAC models, this hybrid effectiveness-implementation trial will evaluate the impact of a proactive enrollment strategy that links smokers seeking LCS with a digital cessation intervention comprising a WEB program and integrated TXT alone and in combination with TTS counseling. Our trial is guided by the RE-AIM framework [70] which provides a systematic way to evaluate the impact of the implementation of public health interventions on five criteria: reach; efficacy/effectiveness; adoption; implementation; and maintenance. This study will focus on reach, effectiveness, and adoption. The primary outcome is biochemically confirmed smoking abstinence at 12 months. The specific aims are as follows: Aim 1 (Effectiveness): to evaluate the comparative effectiveness of WEB + TXT and WEB + TXT + TTS vs a usual care (UC) AAR control group. *Hypothesis 1a:* WEB + TXT + TTS yields higher quit rates than WEB + TXT and UC. *Hypothesis 1b:* WEB + TXT yields higher quit rates than UC; Aim 2 (Implementation): to examine whether proactive enrollment increases treatment use and whether treatment use mediates the relationship between treatment assignment and smoking outcome. *Hypothesis 2a:* Proactive enrollment in WEB + TXT will yield greater levels of treatment use than passive referral to the same interventions in UC. *Hypothesis 2b:* Increases in treatment use will mediate the relationship between treatment group and outcome; Aim 3 (Reach and Adoption): to examine the representativeness of the study sample relative to all smokers screened for LCS eligibility (reach) and the potential for intervention implementation (adoption) among LCS clinics.

Our trial is structured as a practical clinical trial [71] to answer key questions about implementation and effectiveness of direct relevance to decision-makers. The evaluation of interoperable, digital technologies to deliver a sustainable, scalable cessation intervention and to facilitate its integration within clinical practice will add to the growing knowledge base about the overall effectiveness of digital interventions and their role in the healthcare delivery system.

## Methods/design

**Fig. 1:**
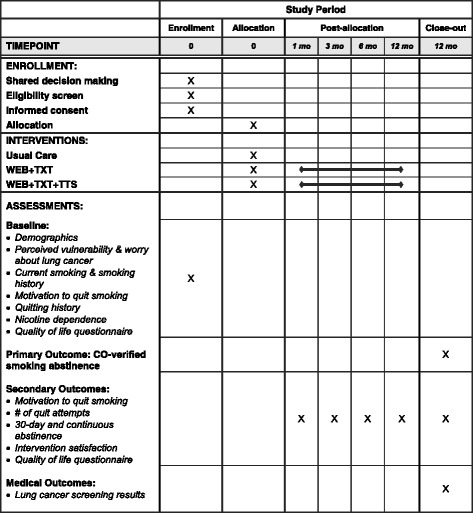
SPIRIT figure

This randomized clinical trial follows the SPIRIT guidelines (see Additional file 1). The study schedule of enrollment, interventions, and assessments is presented in Fig. 1.

### Study setting

This multisite trial will include a mix of healthcare sites serving patients from varied and diverse communities across four states: four sites from the Mayo Clinic Health System; two Mayo Clinic campuses in urban areas; and a destination medical center in Rochester, MN, that serves patients from all 50 states. These sites include a mix of hospital and community centers that are located throughout the US (upper Midwest, Southwest, Southeast) and facilities that are large and small, urban and rural. This variety in study sites will ensure generalizability to other healthcare systems and LCS programs in moderate to large healthcare facilities.

Across study sites, all aspects of LCS and clinical trial management will be facilitated by the Big One Stop Shop (BOSS) Registry, an in-house, Mayo-owned solution that serves as a surrogate for Mayo Clinic’s planned system-wide, single-instance EHR. The BOSS Registry is a WEB system that is highly scalable and used by various groups in research and clinical practice. It will be adapted to store baseline assessment data specific to this trial, house the randomization scheme, and notify the site Study Coordinator when potential participants schedule a shared decision-making visit. The BOSS Registry will also be modified to allow for secure exchange of data with the BecomeAnEX system immediately upon randomization and throughout the study.

### Participants

We will recruit 1650 current smokers who complete a LCS shared decision-making visit. Patients will be eligible regardless of whether it is their first shared decision-making visit or not. Study eligibility criteria are: (1) current smoking (every day/some days in the past 30 days); (2) at least weekly use of the Internet; (3) cell phone ownership with a TXT plan; and (4) willingness to receive study text messages. Exclusion criteria are: current use of cessation medication; participation in tobacco cessation treatment; or failure to sign informed consent.

### LCS eligibility screening and shared decision-making

Patients can self-refer for LCS or be referred by a medical provider. Interest in quitting or in participating in a smoking cessation program are not prerequisites for self-referral or provider-referral. Patients will complete a telephone screening during which demographic information, smoking history, current smoking status, and other data for risk calculation are collected. Eligibility criteria for LCS (per US Preventive Services Task Force) are: (1) age 55–80 years; (2) 30 + -pack/year history of smoking; (3) current smoker or one who has quit smoking within the last 15 years; and (4) asymptomatic (no signs or symptoms of lung cancer) [72]. The Mayo Clinic LCS program also recommends screening to those who are at high risk with a PLCO_m2012_ risk calculation of ≥ 1.34% over the next six years, but who do not meet USPSTF criteria [73]. If the patient is eligible for LCS, a face-to-face shared decision-making session is scheduled during which the risks and benefits of LCS – and the benefits of quitting smoking – are described and patients are helped to make an independent decision about whether to proceed with LCS. Medicare covers payment for LDCT screening for those aged 65–74 years with a 30 + -pack/year history; patients who are no longer smoking must have quit within 15 years. Programs of the Affordable Care Act cover those who meet USPSTF criteria. Private insurance or out-of-pocket payment is required for those who are at high-risk for lung cancer but do not meet CMS or USPSTF criteria.

### Study recruitment and informed consent

The site Study Coordinator will be notified by the BOSS Registry when an eligible, current smoker is scheduled for shared decision-making. At the end of shared decision-making, current smokers who decide to proceed with LCS will be presented with study information. Interested smokers will complete study eligibility screening, sign informed consent, and complete a computerized baseline assessment. Smoking cessation treatment will be initiated immediately following the baseline assessment per the randomization assignment.

### Randomization

Randomization will be automated by computer algorithm and stratified by study site.

### Overview of smoking cessation interventions

All study participants will be given an educational booklet about pharmacotherapy called *Medications to Help You Stop Using Tobacco* [74]. Developed at Mayo Clinic, this booklet provides the rationale and instructions for using medications and describes the mechanisms by which medications work. It is not possible to blind participants to intervention assignment given the nature of the interventions. However, the two experimental arms are standardized and/or delivered according to a protocol.

#### Usual care (UC) control arm

This arm represents tobacco dependence treatment within LCS programs as mandated by CMS. The site Study Coordinator will conduct brief cessation counseling (<3 min). Consistent with the AAR approach [6], this discussion will involve asking about tobacco use, advising the participant about the importance of quitting smoking, and referring the participant to cessation treatment. Referral to treatment in this arm will be passive, consisting of printed information about the digital intervention (BecomeAnEX website and TXT program, details below) and Mayo Clinic TTS services. To facilitate detailed tracking of digital treatment use among participants that follow through with either of these referrals, we will provide a custom URL for the BecomeAnEX website and a study-specific text message enrollment number. This will enable us to identify Arm 1 patients in the BecomeAnEX database and evaluate digital treatment use in a comparable manner across all three treatment arms.

#### WEB + TXT intervention

The site Study Coordinator will conduct brief cessation counseling (<3 min) and then proactively enroll participants in the digital intervention using a study-specific enrollment page on BecomeAnEX. After locating the participant in the BecomeAnEX database using a unique study identifier, the Study Coordinator will assist the participant in completing registration for the WEB and TXT program and provide a brief orientation about both interventions.

The BecomeAnEX website is Truth Initiative’s smoking cessation program that was launched in 2008 [75, 76]. The program was designed around national guidelines for tobacco dependence treatment [43] and principles of social cognitive theory [77]. A national mass media campaign [75] and ongoing online advertising have resulted in over 800,000 registered users since its inception. BecomeAnEX educates smokers about the behavioral and pharmacologic aspects of nicotine addiction and provides tools to enhance self-efficacy for quitting. It guides and supports smokers through a series of interactive components that assist users in selecting a quit date, identifying their smoking triggers, developing coping skills, and selecting a cessation medication. The site also includes a large social network comprising thousands of current and former smokers who interact via several asynchronous communication channels. All user actions are date- and time-stamped and stored in a relational database. BecomeAnEX was extensively pilot tested during development [75] and designed around website usability best practices, making it appropriate for use by older adults [78–81].

The BecomeAnEX TXT program is a fully automated system that has been integrated into the website. The TXT program is also based on social cognitive theory [77]. Messages are tailored around a user-entered quit date and provide general quitting advice, positive reinforcement, motivation, reminders to avoid smoking cues and triggers, and information about nicotine and withdrawal. Keywords (e.g. “CRAVE,” “MORE”) generate on-demand support and information and “STOP” terminates the program at any time. The program delivers three weeks of once-daily cessation text messages regardless of whether a user sets a quit date. When a user sets a quit date, they receive two weeks of twice daily messages pre-quit date and six weeks of 1–3 messages/day post quit date, with periodic messages at cessation milestones (e.g. six months post quit date).

Both the WEB and TXT programs will be customized for delivery to LCS patients. The user will see study information on their individual home page to make clear they have arrived at the correct website. LCS-specific content and videos to reinforce the importance of cessation regardless of LCS results will be added to the website. For the TXT program, we will extend the duration to make it a 12-month intervention to prompt re-engagement with the WEB components and to remind patients of their one-year LCS appointment. Participants will receive four messages per month beyond the standard program described above.

#### WEB + TXT + TTS intervention

Initiation of the WEB + TXT intervention in this arm is identical to the approach described above. Unique to this treatment arm is a required session with a TTS before LCS. During this session, the TTS will conduct an assessment, formulate a treatment plan, and provide counseling, education, and arrange for follow-up. TTS counselors will be able to view real-time utilization data from BecomeAnEX through the BOSS Registry, already part of their routine workflow. They will know which elements of the website the participants have used/not used and will be able to incorporate this information into the counseling session. The TTS will utilize motivational interviewing skills and strategies to create momentum for quitting and actively engage the patient. TTS counseling incorporates information gathered from an assessment to collaboratively develop an individually tailored treatment plan that consists of cognitive and behavioral strategies and pharmacological treatment. An interactive patient treatment guide will be used to standardize the educational and counseling content. At the conclusion of this initial session, the TTS will schedule four additional sessions.

#### Lung cancer screening

Low dose computed tomography (LDCT) LCS will be conducted in the Mayo Lung Screening Program. All sites are designated as American College of Radiology (ACR) LCS centers. The parameters of the screening at each participating site are done in accordance with ACR and CMS requirements [82]. The screening is done with the patient lying in the scanner with arms overhead and accomplished in a single breath-hold in < 15 s. The CT is read by a thoracic radiologist who has received ACR accreditation and read using the Lung-RADS (lung imaging reporting and data system) standardized lung nodule reporting scheme as required by ACR [83]. The program collects and submits data to the ACR registry for each LDCT LCS performed. Every patient receives a letter with recommendations and their scan report, which are also sent to their designated practitioner. The next annual screening CT is recommended for patients with a normal scan or those with one or more nodules < 6 mm; interval follow-up is provided for nodules < 10 mm. Patients with nodule(s) ≥ 10 mm or adenopathy are referred to a pulmonologist associated with the Mayo Lung Screening Program and pursued in accordance with published guidelines using a multidisciplinary approach.

### Assessments

Assessments will occur at baseline and one, three, six, and 12 months post randomization. Follow-ups will be conducted by telephone by research staff who are not blind to treatment assignment but who do not have any role in intervention delivery. We will pilot test measures with 8–10 LCS patients (not participants) to ensure wording and administration are appropriate for older adults [84]. We expect at least 80% follow-up at 12 months based on previous research by our team [29, 85, 86] and retention of > 90% in previous Mayo Clinic LCS studies [87]. To maximize follow-up, we will: (1) provide clear information about the study at the outset, including expectations for follow-up data collection; (2) reimburse participants with graduated incentives for all follow-up data collection, including a separate incentive for CO testing at 12 months; and (3) emphasize the importance of survey completion regardless of smoking status. If follow-up rates are lower than expected, we will consider implementing additional modes of follow-up (e.g. online survey) used successfully by our team at minimal cost.

#### Baseline variables

To characterize the sample and examine moderators of treatment effectiveness, the baseline assessment will assess: demographics (age, sex, ethnicity, race, marital status, education, number of household tobacco users); perceived vulnerability and worry about lung cancer (personal risk of developing cancer, their likelihood compared to other smokers, and their level of worry about getting lung cancer); current smoking and smoking history (smoking frequency and rate, lifetime and past 30-day use of other tobacco products and e-cigarettes); motivation to quit smoking will be measured with the Readiness Ladder [88, 89] and an item regarding confidence in quitting; quitting history, including past year quit methods; nicotine dependence will be measured with the Fagerström Test for Nicotine Dependence [90].

The following metrics will be extracted from the BOSS Registry for all patients that are determined eligible for LCS: age; education; race/ethnicity; body mass index; referral source; and smoking status. We will compare patients eligible for LCS to study participants on these characteristics to evaluate representativeness of the study sample.

#### Process variables

Treatment utilization will be assessed via self-report at each follow-up and automated tracking metrics extracted from BecomeAnEX and the BOSS Registry. At each follow-up, participants will be asked about their use of any cessation methods. The following metrics will be extracted from BecomeAnEX: total time spent logged into the website; number of return visits; use of the static content and videos; use of interactive components/tools; number of replies to interactive text messages; unsubscribe rates; modal day of unsubscribe; number of days enrolled in TXT; and number of text messages received. The following metrics will be extracted from BOSS regarding completion of TTS counseling: number and duration of completed TTS counseling sessions; tobacco use status; confidence and importance for quitting or maintaining abstinence; medication use; cravings/urges and management strategies.

TTS intervention fidelity will be monitored throughout the study via review of digital audio recordings. Audio review will be conducted using a fidelity measure based on the Mayo Clinic treatment model including items adapted from the Motivational Interviewing Treatment Integrity Code [60].

#### Primary outcome

The primary outcome is biochemically confirmed (carbon monoxide [CO]) abstinence at 12 months post randomization. All participants who self-report seven-day abstinence at the 12-month follow-up will be invited to provide a breath sample for CO testing. Expired alveolar CO concentrations of ≤ 10 ppm will be considered verification of abstinence [91]. Participants who decline testing will be considered smokers.

#### Secondary outcomes

Other smoking-related outcomes will include change in motivation to quit, number of quit attempts, and 30-day and continuous abstinence measured at each follow-up [92]. Intervention satisfaction in all three arms will be measured with an adapted version of the Client Satisfaction Questionnaire [93]. We will assess overall satisfaction and perceived helpfulness, whether the intervention met their expectations (1 = not at all, 5 = very much), and whether they would recommend it to a friend (yes/no). We will include items that address usability of the WEB and TXT interventions by modifying measures from the System Usability Scale (all measures 1 = strongly disagree, 5 = strongly agree): whether they found them unnecessarily complex; whether they found them easy to use; whether they felt they would need the support of a technical person to be able to use them; whether they felt very confident using them; whether there was too much inconsistency in them; and whether they would imagine that most people would learn to use them very quickly [94]. Quality of life will be measured at baseline and 12-month follow-up with the Smoking Cessation Quality of Life Questionnaire [95, 96] and will be assessed with a single item 10-point rating scale at one-, three-, and six-month follow-ups.

#### Medical outcomes

BOSS provides the ability to capture radiology results of LDCT of patients with lung nodules such as Lung-RADS, follow-up procedures, diagnosis of lung cancer, vital status, and other concurrent diagnoses. Follow-up LCS status will be determined using data from the BOSS Registry at one year (all participants) and two years (~80% participants).

### Data monitoring

At the start of the study, a Data Safety Monitoring Committee will be established, comprising the Principal Investigators, Data Analyst, Biostatistician, Technical Lead, and Project Manager. Every two weeks the committee will review participant accrual rate, participant drop-out and the reasons for drop-out, target enrollment status, major and minor problems related to treatment assignment, and serious adverse event reports.

This is a minimal risk study for which no Data Safety Monitoring Board is needed. Standard clinical trial operating procedures for crisis management will be in place throughout the trial. Study staff will monitor and report serious adverse events.

### Power calculation

Positive (i.e. abnormal) LCS findings increase the odds of abstinence [6, 97–99]. Therefore, to be conservative in our sample size calculations, efficacy estimates for UC are based on data from patients in previous trials with negative screening results. Among patients with negative findings in the National Lung Screening Trial, one-year abstinence rates are in the range of 9.7% [100] to 12.6% [97]. In the Danish Lung Cancer Screening Trial [98], patients received < 5 min of counseling from a nurse and 30-day abstinence at one-year follow-up was 11.4% among patients with no significant LCS findings. Cox et al. [101] reported seven-day abstinence at one year of 13%. Brief counseling for smoking cessation (<3 min) yields six-month abstinence rates of 13.4% [43]. Therefore, we conservatively estimate that biochemically verified abstinence at 12 months for UC will be 10%.

Efficacy estimates for WEB + TXT are based on several converging lines of evidence: (1) two meta-analyses have reported statistically significant effects of interactive and individually tailored interventions compared to usual care or written self-help (relative risk [RR] = 1.48, 95% confidence interval [CI] = 1.11–2.78 [30]; RR = 2.10, 95% CI = 1.25–3.52 [33]; (2) a meta-analysis by Webb et al. [102] found that the use of TXT in Internet interventions had large effects on behavior change (d = 0.81, k = 4); (3) combined Internet and TXT interventions yielded six-month sustained abstinence of 17.1% in Borland et al. [103] and 12-month repeated point prevalence abstinence of 20% in Brendryen et al. [46]. Therefore, we conservatively estimate that biochemically verified abstinence at 12 months for WEB + TXT will be 16%.

Efficacy estimates for WEB + TXT + TTS are also based on several lines of evidence: (1) abstinence reported by Fiore et al. [43] for treatment duration of 31–90 min (comparable to the TTS visit that will occur before LCS) was 26.5% (95% CI = 21.5–31.4); (2) among more than 6000 patients who received TTS counseling in the NDC outpatient clinic, seven-day point prevalence abstinence at six months under intention-to-treat (ITT) analysis was 28.1% (95% CI = 27.7–30.1) [60]; (3) in a pilot study of LCS patients who completed a TTS visit before LCS, seven-day abstinence at six months (ITT) was 34% [64]; (4) previous studies of combined Internet and TTS counseling interventions have yielded quit rates (30-day point prevalence abstinence under ITT) of 21.5% [29] to 53% [69] with a strong dose-response relationship for the number of TTS sessions. Therefore, we conservatively estimate that biochemically verified abstinence at 12 months for WEB + TXT + TTS will be 24%.

For pairwise treatment comparisons, we will use the Holm–Šídák approach to account for multiple comparisons [104]. The three pairwise comparisons will be performed with two-tailed *p* values obtained which are not adjusted for multiple comparisons. These *p* values will be ranked from smallest to largest. If the smallest *p* value is > 0.0167 we will conclude that none of the comparisons is statistically significant. If the smallest *p* value is ≤ 0.0167 this comparison will be considered statistically significant and the second smallest *p* value will be evaluated. If the second smallest *p* value is > 0.025, we will conclude that the final two comparisons are not statistically significant. If the second smallest *p* value is ≤ 0.025, this comparison will be considered statistically significant and the final *p* value will be evaluated using *p* ≤ 0.05 to denote statistical significance. For our hypothesized abstinence rates, a total sample-size of 1650 (550/group) will provide statistical power ≥ 99% for the overall comparison across treatment groups and > 85% for each of the three pairwise treatment comparisons.

### Statistical analyses

Unless otherwise specified, analyses use an ITT approach that analyzes individuals by treatment assignment. We will use descriptive statistics to summarize demographic data, tobacco use history, and other baseline characteristics. Summaries will be generated for the entire sample and by treatment arm. Given the randomized design, we will not assess for significant baseline treatment differences. With the exception of the stratification variable, no covariate adjustment will be included in the primary analysis. For all exploratory analyses, two-tailed *p* values will be reported with no adjustment for multiple comparisons. In all cases, findings will be reported using point estimates and corresponding 95% CIs. Model assumptions for each analysis will be validated and transformations or non-parametric methods will be utilized as appropriate.

#### Aim 1 (Effectiveness) analyses

The primary outcome of biochemically confirmed point-prevalence abstinence at 12 months post randomization will be analyzed using ITT. The primary analysis of the smoking outcomes will be performed using logistic regression. Separate analyses will be performed for each abstinence endpoint. For the logistic regression analyses, the dependent variable will be smoking abstinence and the independent variable will be treatment (WEB + TXT + TTS vs WEB + TXT vs UC). Study site will be included as a covariate in the primary analyses. We hypothesize that WEB + TXT + TTS will yield higher quit rates than WEB + TXT and UC and that WEB + TXT will yield higher quit rates than UC. To test these hypotheses, all pairwise comparisons of the three treatment groups will be performed. For the primary analysis of smoking abstinence outcomes, any individual who misses a visit will be classified as smoking for that assessment. This approach is used because the data are assumed to be missing not at random (MNAR). However, this approach is sensitive to differential attrition across treatment groups and tends to overestimate the precision of the estimate of the treatment effect. Therefore, to supplement primary analysis we will perform sensitivity analysis using multiple-imputation based on pattern-mixture models under the MNAR assumption (SAS/STAT version 13.2, PROC MI and PROC MIANALYZE). Multiple imputations of missing abstinence outcomes will be imputed using the logistic regression method, with the outcome adjusted by modifying the log odds ratios (OR) [105, 106]. Since the OR relating missingness to smoking is not known, we will conduct a sensitivity analysis to assess its impact on the estimate and significance of the treatment effects.

We will examine potential moderators (e.g. screening result, gender, age) by analyzing interactions between treatment and selected variables. We will examine treatment/moderator interaction term on outcomes after entering main effects. We will also explore moderators of the relationship between treatment assignment and treatment utilization.

#### Aim 2 (Implementation) analyses

**Fig. 2:**
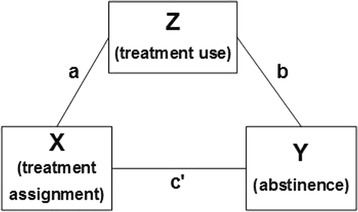
Mediational model

First, we will examine treatment uptake and engagement via self-reported treatment use (behavioral or pharmacologic) and automated tracking data from the BecomeAnEX WEB and TXT program at each follow-up interval. We will test whether proactive enrollment in treatment yields higher levels of treatment uptake and engagement than AAR UC using descriptive statistics. We hypothesize that overall utilization will be higher for participants proactively enrolled in WEB + TXT compared to participants provided passive referrals to the same interventions in UC. Next, we will examine whether treatment engagement mediates the treatment-outcome relationship. To assess whether treatment engagement is predictive of improved abstinence outcomes and whether treatment engagement mediates the treatment effect, we will perform a series of additional logistic regression analyses following the framework described by Baron and Kenny [107]. The Aim 2 analytic plan is depicted in Fig. 2.

Here, X is the exogenous variable (treatment), Z is the hypothesized mediator (treatment engagement), Y is the outcome (abstinence at month 12), a is the regression path between treatment and the mediator, b is the regression path between the mediator and the outcome, and c’ is the regression path between treatment and the outcome controlling for the effect of the mediator.

To test mediation hypotheses, we will use the approach described by MacKinnon and Dwyer for binary mediator and outcome variables [108]. We will establish mediation as follows: (1) logistic regression will assess whether the effect of treatment assignment on abstinence is statistically significant; (2) logistic regression will assess whether the effect of treatment assignment on treatment use is statistically significant; (3) multivariable logistic regression will determine whether treatment use is statistically associated with abstinence after adjustment for treatment assignment; and, (4) we will assess whether the effect of treatment assignment on outcome is significantly attenuated in magnitude relative to step 1.

We will compare total and incremental costs to implement each intervention from a payer perspective. Implementation costs of the two interventions will be estimated using average market values for salaries of the additional staff needed to perform the TTS counseling and market costs of the digital intervention. If a treatment effect is observed, we will assess cost per quit and incremental cost per quit. Cost-effectiveness of the two proposed study interventions compared to usual care will be estimated by employing a Markov Model [109, 110]. Hypothetical cohorts representing the U.S. population will be simulated through long-term screening. Disease progression will be modeled with transition probabilities of developing lung cancer. Model parameters not directly measured in the proposed trial will be supplemented with data from the literature, including the publicly available Lung Cancer Policy Model of the Cancer Intervention and Surveillance Modeling Network [111–113]. Model complexity specific to our modeling environment will be determined by available data for the model parameters. A simplistic design not incorporating smoking status (current vs smoker) will be used as the model basis. Smoking status will be incorporated if identified data prove to make this feasible. If not feasible, the simplistic model design will serve as a crucial starting point for further analyses in the future. Sensitivity analyses will be conducted to determine the impact of changes in key model inputs.

#### Aim 3 (Reach and Adoption) analyses

Reach will be evaluated as the representativeness of the enrolled sample relative to all smokers that are assessed for LCS eligibility. We will compare the enrolled sample to those that are assessed for LCS eligibility on available demographic, smoking history, and medical history variables using Chi-square tests and T-tests.

Adoption will be assessed through a mixed mode quantitative/qualitative approach. We will conduct an enterprise-wide survey of LCS program directors and other key informants (e.g. nursing staff, office managers) in all LCS facilities within Mayo Clinic. The survey will gather detailed information about the barriers that would impede adoption of the digital and combined digital/clinical treatments evaluated in this study, as well as the organizational processes or resources that would facilitate their adoption and implementation. We will also conduct structured interviews with members of the Mayo Clinic Lung Screening Program to gather in-depth information about the feasibility of these treatment approaches and site-specific recommendations about their implementation.

### Protocol amendments

Changes to the research protocol will be reviewed by the Institutional Review Board (IRB). Amendments will be made to the trial registry as necessary.

### Confidentiality

The data management will be governed by standard procedures with regard to data security and access. All analyses are logged with respect to IRB authorization, accounting information, principal and secondary investigators, statistician, and data analyst involved in the analysis.

BecomeAnEX has stringent privacy and security policies in place which include a comprehensive privacy policy, strong encryption used in the transmission of all health-related information, electronic access controls on all personally identifiable information, and industry standard network protection. All information collected via the Internet will be kept secure in transit using the Secure Socket Layer (SSL) protocol. Both the BecomeAnEX WEB and TXT systems are cloud hosted platforms and have security audits performed annually. All communications between users and BecomeAnEX.org, and between BecomeAnEX.org and the TXT system, are sent securely via https. Both systems maintain offline backups stored in geographically separate areas for disaster recovery. All data transferred to and from the study sites will be de-identified and uploaded using a secured transfer protocol. All data will be stored in a database subject to both physical and electronic protection. The BecomeAnEX Privacy Policy is available at http://www.becomeanex.org/privacy-policy.php and will be in effect for all participants that register on the website.

### Dissemination

Trial results will be posted on ClinicalTrials.gov, published in peer-reviewed journals, and presented at national conferences.

## Discussion

Several key practical and operational issues warrant additional discussion. Here, we summarize key aspects of our implementation approach and the rationale underlying our decisions.

### Why is pharmacotherapy not provided?

This study is designed to examine the effectiveness of interventions as delivered in LCS programs; pharmacotherapy is not typically delivered in this setting. We have aimed to strike a balance between maximizing generalizability while retaining critical elements of internal validity. All patients will be provided with a booklet that provides a rationale for medication use and describes first-line medications for smoking cessation. The WEB and TXT interventions also include information about and support for use of medication. We are interested in the extent to which each intervention arm promotes medication use, which is a dependent variable that will be examined in Aim 2.

### Why not compare proactive enrollment in a digital intervention to proactive enrollment in a telephone quit line?

Although quit lines are the typical referral resource, proactive enrollment to them is not yet standard of care. Our decision is also rooted in an inability to track uptake and engagement of quit line resources once referred. This study will be recruiting from LCS programs in four different states that may provide different quit line services at any given time. Coordinating data exchange with quit lines is beyond the scope of this funding mechanism. A digital intervention may be delivered with complete fidelity and monitoring, regardless of patient location.

### Given high quit rates for TTS counseling, why not evaluate the additional impact of a digital intervention added to TTS counseling?

Our decision was based on costs and the ease of implementation with LCS programs. TTS counseling does yield higher quit rates but at significantly higher cost [34, 35] and with additional considerations about how it would be incorporated into LCS workflow. Proactive enrollment in digital cessation treatments can be automated via an EHR and implemented at significantly lower cost, making it potentially more appealing for the majority of LCS settings. Aim 3 analyses will specifically address these important questions about adoption.

This study employs innovative technologies and integrated tobacco treatment modalities to promote sustainable behavior change among smokers undergoing LCS. There are few examples of fully integrated clinical and digital interventions where uptake and ongoing engagement in technology-based interventions are explicit targets of counseling. The approach in this study has the potential to be a model for not only LCS programs, but for any medical practice that treats illnesses caused by tobacco dependence. In addition, our study builds on substantial evidence for the effectiveness of proactive enrollment in cessation treatment and leverages the availability of an EHR to automate enrollment and provide real-time patient data back to the provider. EHR-supported proactive enrollment in cessation treatment has not been tested within LCS settings. There are few studies that have initiated cessation treatment immediately after shared decision-making and before LCS. This approach is designed to communicate to smokers that cessation is a priority regardless of LCS results.

Results from this study will inform the key characteristics and components of an effective smoking cessation intervention delivered in the LCS setting. Our project addresses proactive vs passive enrollment in treatment as a key element of program implementation, which has important implications for treatment uptake and engagement. The study involves proven digital (WEB and TXT) and clinical (TTS) intervention strategies and a novel approach to integrate them to address implementation challenges within the LCS setting. Our focus on the seamless integration of clinical information and behavioral health technologies aligns with federal health IT priorities in improving population health [57] and is consistent with recent guidelines for the delivery of cessation treatment in the LCS setting [19]. Our approach will inform the integration of a clinically feasible, cost-effective, and sustainable approach to tobacco dependence treatment into healthcare systems as part of usual practice.

### Trial status

Study recruitment was underway at the time of manuscript submission. The trial began recruiting in August 2017.

## Additional file

Additional file 1: SPIRIT Checklist. (DOC 121 kb)

## Abbreviations

AAC: Ask–Advise–Connect
AAR: Ask–Advise–Refer
ACR: American College of Radiology
BOSS: Big One Stop Shop
CMS: Centers for Medicare and Medicaid Services
CO: Carbon monoxide
EHR: Electronic health record
IQR: Interquartile range
IRB: Institutional Review Board
ITT: Intent to treat
LCS: Lung cancer screening
LDCT: Low dose computed tomography
Lung-RADS: Lung imaging reporting and data system
MNAR: Missing not at random
NDC: Nicotine Dependence Center
NRT: Nicotine Replacement Therapy
PROC: Procedure
RE-AIM: Reach Effectiveness Adoption Implementation Maintenance
SSL: Secure socket layer
TTS: Tobacco treatment specialist
TXT: Text messaging
UC: Usual care
URL: Uniform resource locator
